# One-Step Process of Mixed Oleic Acid Esters and Its High Temperature Lubrication Properties in Bentonite Gelling Suspension

**DOI:** 10.3390/gels8100678

**Published:** 2022-10-20

**Authors:** Xincheng Bao, Cunfa Ma, Fengshan Zhou

**Affiliations:** 1 Beijing Key Laboratory of Materials Utilization of Nonmetallic Minerals and Solid Wastes, National Laboratory of Mineral Materials, School of Materials Science and Technology, China University of Geosciences (Beijing), No. 29 Xueyuan Road, Haidian District, Beijing 100083, China; 2 Ningxia Baofeng Group Co., Ltd., Office Building, No. 19, International Trade City, Lijing Street, Xingqing District, Yinchuan 750003, China

**Keywords:** water-based drilling fluids, esterification, lubricant, temperature tolerance

## Abstract

In recent years, with the increase in requirements for horizontal wells, ultra-high depth wells, small wells and branching wells, it has become increasingly important to deal with the conflict between drilling safety and bottomhole friction. In order to meet the requirements of complex boreholes and deepwater drilling processes, it is crucial to improve the performance of ester-based lubricants. Oleic acid esters are relatively stable and have high lubricity at low temperature, however, these can be hydrolyzed at high temperature. However, the structure of carboxylic acids and alcohols can significantly affect the performance of synthetic esters. In order to solve the problem of balancing the high-temperature performance and low temperature performance of oleic acid esters with different structures, we propose a new oleic acid esterification process. After mixing methanol and ethylene glycol, it is reacted with oleic acid, and the mixed oleate prepared is named MEO-21, and the optimal esterification conditions are obtained as follows: the reaction time is 3 h, the reaction temperature is 150 °C, and concentrated sulfuric acid is the catalyst. MEO-21 not only achieves an extreme pressure lubrication coefficient reduction rate (*Δf*) of 86.57% at room temperature, but maintains a stable performance after hot rolling at high temperatures. Hot rolling at 150 °C for 16 h, *Δf* was 85.25%, hot rolling at 180 °C for 16 h, *Δf* was 89.56%. MEO-21 was used as a base oil with other industrial by-product oils to compound and produce a high-temperature-resistant lubricant that was named L-541, L-541′s *Δf* was 90.39% at room temperature. L-541 was hot-rolling at 120 °C, 150 °C and 180 °C for 16 h, the *Δf* was stabled at 89%.

## 1. Introduction

In recent years, with the increase in requirements for horizontal wells, ultra-high deep wells, small wells and branch wells, the conflict between drilling safety and bottom friction has become increasingly important [1]. Compared with oil-based drilling fluids, water-based drilling fluids have the advantages of environmental protection and low cost, and are the current and future main applications of the drilling fluid system, but its lubrication performance is poor, which significantly restricts its promotion and the use of complex structural wells such as large displacement wells and horizontal wells.

Lubricants are added as additives to water-based drilling fluids to improve lubrication and minimize friction [2]. Highly lubricant drilling fluids can also increase the mechanical drilling speed and significantly save costs [3], the system is environmentally friendly, cost-effective and as lubricant as the oil base [4]. A very small amount of lubricant is sufficient to provide sufficient mud lubricity, lubricants as low as 1% reduce the torque by 20% [5], while the average optimal concentration of lubricating oil is less than 3% [5,6,7].

Mineral oil and vegetable oil are the most widely used drilling fluid lubricants [8]. Mineral oil is not easily degradable, which poses a great threat to the environment. Moreover, mineral oil has strong fluorescence, which will interfere with geological logging [8,9,10]. Vegetable oils derived from plants are biodegradable and renewable, a property that encourages the exploration of their applications in drilling fluids [7,11,12,13]. Vegetable oil molecules have good lubricity because of the existence of polar groups in vegetable oil molecules, which can form adsorption films on metal and rock surfaces. As argued in Ref. [14], however, vegetable oils are extremely hydrolyzed, resulting in an increase in viscosity. Since most modern exploration requires a focus on deep formations, where harsh conditions such as high temperatures, salinity, and pressure exist, their use in oil drilling is also limited by poor cold habits, low thermal stability and oxidation stability [15].

The research content mainly focuses on the vulcanization or esterification of the fatty acid of vegetable oil as raw material, so as to improve the lubricity of the base mud and reduce the foaming rate [16]. The esterification or transesterification is the process of changing the chemical structure of vegetable oils by reacting vegetable oils or synthetic fatty acids with alcohols [17,18]. The structure of carboxylic acids and alcohols can significantly affect the performance of synthetic esters [19]. Oleate is relatively stable at low temperatures and has high lubricity, but it can be hydrolyzed at high temperatures, and people cannot eliminate the hydrolysis of esters at high temperatures and excess hydroxyl conditions, but can only reduce it [20]. To meet the requirements of complex borehole and deep water drilling processes, improving the performance of ester-based lubricants is critical, especially in sensitive marine areas [21,22]. However, polyfunctional alcohols containing more hydroxyl groups, such as neopentyl glycol, trimethylolpropane, and pentaerythritol, tend to reduce their molecular flexibility, thereby increasing the kinematic viscosity of synthetic esters [23]. The viscosity of an ester can be reduced by reducing its relative molecular mass and increasing its molecular flexibility [24], and a lower carbon number alcohol and oleic acid reaction can be used to obtain a lighter molecular weight oleate. Therefore, in this study, the esterification of oleic acid and methanol, the esterification of oleic acid and ethylene glycol, and the optimal esterification process were obtained by analyzing the characteristics of the esterification products, so that a completely new esterification process is proposed: methanol and ethylene glycol are mixed and reacted with oleic acid, under experimental conditions which were designed to obtain the optimal process for mixed esterification. This method not only solves the problem of balancing the high temperature performance and low temperature performance of oleate with different structures, but also simplifies the preparation process, and the product has a high cost performance. The esterified product is not easily hydrolyzed either at room temperature or after hot rolling at high temperatures and has excellent lubricity. The resulting high performance mixed oleate is then compounded with other industrial by-product oils to obtain a high-performance liquid lubricant with excellent lubrication performance at room temperature and high temperature, small density change, low fluorescence, and non-toxicity.

## 2. Results and Discussion

### 2.1. Oleic Acid Esterification with Methanol

**Figure 1:**
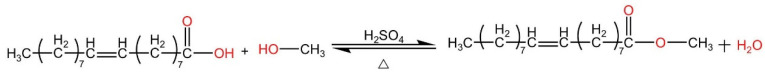
Equation for the esterification of oleic acid with methanol.

The equation for the esterification of oleic acid with methanol is shown in Figure 1 below. Factors affecting the reaction are the reaction temperature, reaction time and catalyst. The catalysts used in this experiment are concentrated sulfuric acid, sodium hydroxide and sulfamic acid, which are the most commonly used for alcohol oil esterification, and the esterification results of oleic acid and methanol are mainly explored at different temperatures, different catalysts, and different reaction times.

The reaction time of this reaction is generally 2~7 h, the reaction temperature is 60~110 °C, and the catalyst addition is 1~3%.

Esterification rate = (pre-reaction acid value − post-reaction acid value)/pre-reaction acid value × 100% [25]. The calculation method of MT quality is as follows:

M_MT_ = (S·M·M_1_·10^−3^)/(M_2_) (1)

In the formula:

S—acid value of oil and grease (mg KOH/g);
M—mass of grease (g);
M_1_—MT molar mass (g/mol);
M_2_—KOH molar mass (g/mol).

The oleic acid mass is set to 100 g, and according to the formula (1), the calculated amount of methanol mass used was 4.24 g. In order to ensure the complete reaction of oleic acid during the experiment, the excess methanol reaction is used, and hence the methanol increase is set to 8 g. After the end of the experiment, turn off the condensing device and continue to heat for 10 min to ensure that the methanol is completely volatilized.

#### 2.1.1. Effect of Catalysts on Esterification

**Figure 2:**
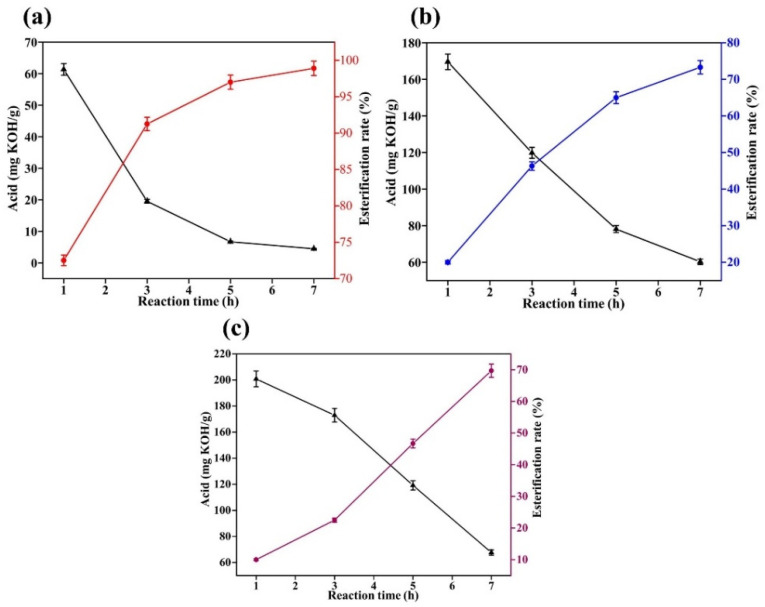
Catalytic esterification results of different catalysts (**a**): concentrated sulfuric acid; (**b**): sodium hydroxide; and (**c**): sulfamic acid.

The reaction temperature of oleic acid and methanol esterification is 60~120 °C, and the minimum temperature of 60 °C is determined as the reaction temperature of the experiment, and the catalyst increase is 1% of oleic acid. To investigate the effect of different catalysts on the catalytic low temperature reaction of oleic acid with methanol, the acid value of the system was measured at half-hour intervals and the results of the experiment are shown in Figure 2.

Figure 2 shows the results of concentrated sulfuric acid, sodium hydroxide and sulfamic acid as catalysts to catalyze the esterification of oleic acid and methanol under low temperature conditions. We can see that the concentrated sulfuric acid catalytic effect is better than the sodium hydroxide and sulfamic acid catalytic effect. When catalyzed by concentrated sulfuric acid, the esterification of oleic acid was already approximately 73% by one hour into the reaction, and by three hours into the reaction, the esterification was over 90%. In contrast, sodium hydroxide and sulfamic acid were less effective in catalysis, with the esterification rate at approximately 40% after 3 h of reaction. This shows that concentrated sulfuric acid can be a good catalyst for the esterification of oleic acid with methanol at low temperatures.

#### 2.1.2. Effect of Temperatures on the Esterification Reaction

**Figure 3:**
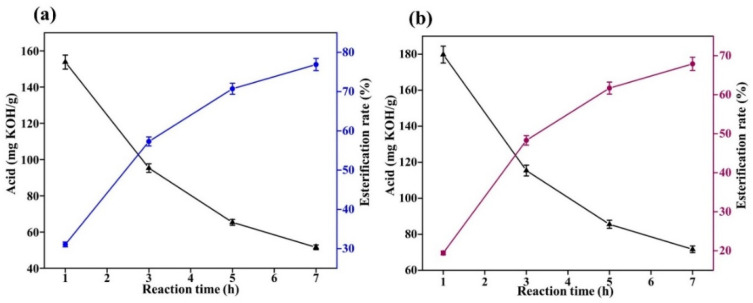
Esterification results of methanol with oleic acid at 90 °C (**a**): sodium hydroxide; and (**b**): sulfamic acid.

**Figure 4:**
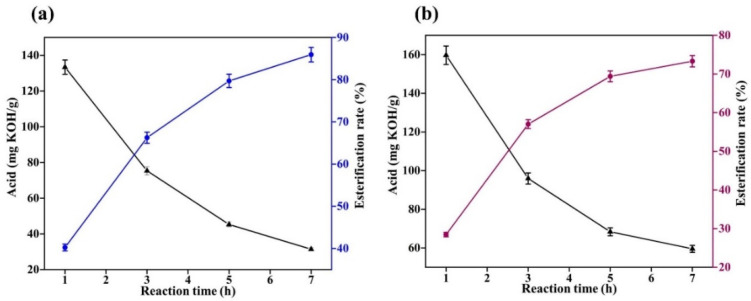
Esterification results of methanol with oleic acid at 120 °C (**a**): sodium hydroxide; (**b**): sulfamic acid.

Under low-temperature conditions, concentrated sulfuric acid can be used as a good catalyst to catalyze the esterification of oleic acid and methanol, and the catalytic effect of sodium hydroxide and sulfamic acid is poor. However, in the production process of the enterprise, it is found that concentrated sulfuric acid has greater corrosiveness for the metal reactor, and long-term use will cause greater corrosion to the reactor. Therefore, when the experimental temperature increases, sodium hydroxide and sulfamic acid should be selected as catalysts, the temperature was designed at 90 °C and 120 °C, and the acid value of the system is determined every half an hour. The experimental results are shown in Figure 3 and Figure 4.

As Figure 3 shows temperature comparisons at 60 °C and 90 °C, both sodium hydroxide and sulfamic acid accelerate the esterification reaction rate of oleic acid with methanol. After 3 h of reaction, the sodium hydroxide catalyzed esterification was close to 60%, while the sulfamic acid esterification was lower, at approximately 50%.

As can be seen from Figure 4, at a temperature of 120 °C and 3 h of reaction, the esterification rate was close to 70% under sodium hydroxide catalysis and less than 60% under sulfamic acid catalysis.

Taking all factors into consideration, the esterification of methanol with oleic acid should be carried out with the better catalytic effect of concentrated sulfuric acid as the catalyst at a temperature of 60 °C. This esterification reaction requires a lower temperature, is safer to operate and has a higher esterification rate.

### 2.2. Oleic Acid Esterification with Ethylene Glycol

**Figure 5:**
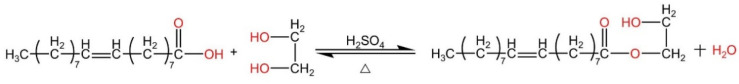
Equation for the esterification of oleic acid with ethylene glycol.

The equation for the esterification of oleic acid with ethylene glycol is shown in Figure 5 below, and the reaction influencing factors are the reaction temperature, reaction time, and catalyst. The catalysts used in this experiment are the concentrated sulfuric acid, sodium hydroxide and sulfamic acid, which are the most commonly used in esterification, and the esterification results of oleic acid and methanol are mainly explored at different temperatures, different catalysts and different reaction times.

The reaction time for the esterification of oleic acid and ethylene glycol is generally 2~7 h. The reaction temperature is 160 °C~200 °C and the catalyst addition is 1~3%. The mass of oleic acid was set at 100 g. The mass of ethylene glycol used was calculated according to equation (1) to be 7.99 g, to ensure the complete reaction of oleic acid during the experiment, excess ethylene glycol was used for the reaction and the amount of ethylene glycol added was set to 15 g. After the end of the experiment, the condensing device was turn off and heating was continued for 10 min to ensure that the methanol was completely volatilized. After the experiment, the esterification rate of oleic acid was calculated.

#### 2.2.1. Effect of Catalysts on Esterification

**Figure 6:**
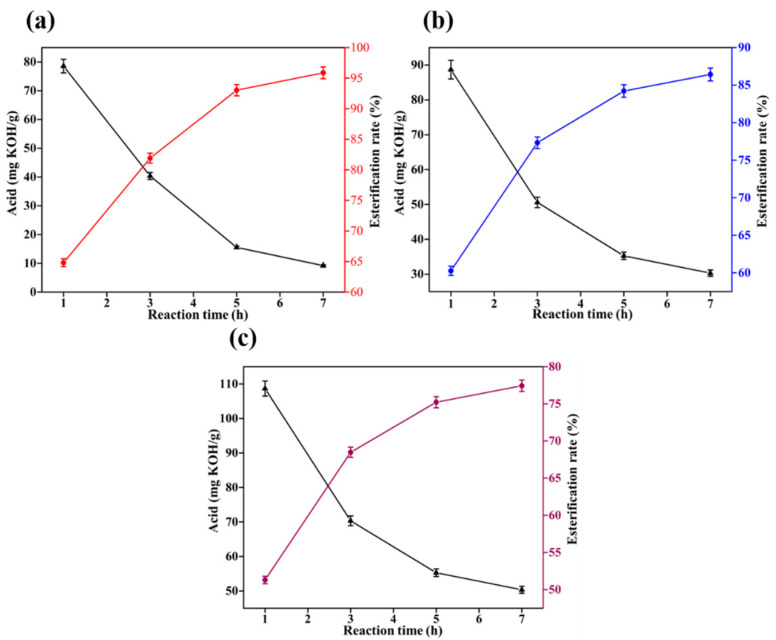
Catalytic esterification results of different catalysts (**a**): concentrated sulfuric acid; (**b**): sodium hydroxide; and (**c**): sulfamic acid.

The reaction temperature of oleic acid with ethylene glycol esterification is 160 °C~200 °C, the minimum temperature of 160 °C is determined as the reaction temperature of the experiment, and the catalyst is increased to 1% of oleic acid. To explore the effects of different catalysts on the catalytic low-temperature reaction between oleic acid and ethylene glycol, the acid value of the system was determined every half an hour, and the experimental results are shown in Figure 6.

From Figure 6, it can be seen that when oleic acid is esterified with ethylene glycol, at 160 °C, the catalytic effect of concentrated sulfuric acid and sodium hydroxide is close, and when the reaction is 3 h, the esterification rate is close to 80%. Upon continuing the reaction for 5 h, the esterification rate increased to approximately 85%. However, the catalytic effect of sulfamic acid obtained was poor. When the reaction time was 5 h, the esterification rate obtained was less than 70%. Therefore, for the esterification catalyst of oleic acid and glycol, we can choose concentrated sulfuric acid or sodium hydroxide.

#### 2.2.2. Effect of Temperatures on the Esterification Reaction

**Figure 7:**
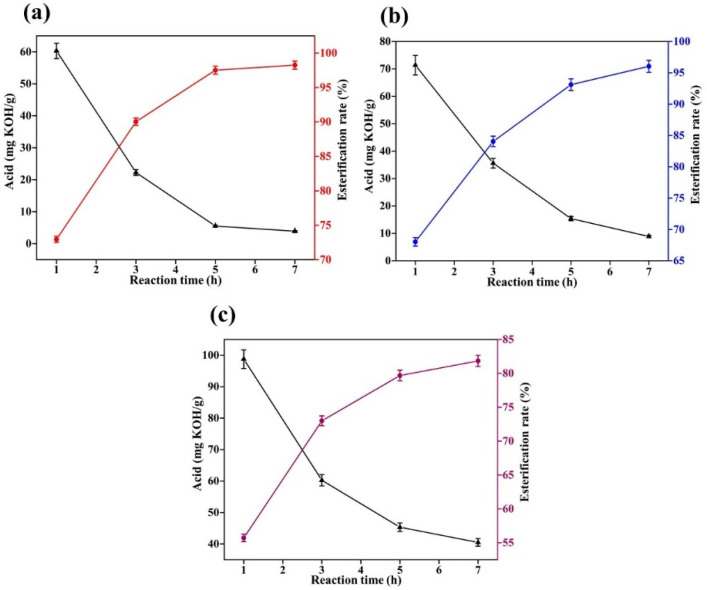
Results of catalytic esterification with different catalysts at 180 °C: (**a**): concentrated sulfuric acid; (**b**): sodium hydroxide; and (**c**): sulfamic acid.

The above experiments found that at 160 °C, the concentrated sulfuric acid and sodium hydroxide have a similar catalytic effect on oleic acid and ethylene glycol, the reaction is up to 3 h, and the esterification rate is approximately 80%. In order to further explore the effect of temperature on the esterification reaction, the reaction temperature was increased to 180 °C, the reaction was continued, the acid value of the system was tested every half hour, and the experimental results are shown in Figure 7.

From Figure 7, it can be concluded that increasing the temperature accelerates the esterification reaction rate. At 180 °C, concentrated sulfuric acid was catalyzed for 3 h, and the esterification rate of oleic acid and ethylene glycol reached 90%. Under the catalysis of sodium hydroxide, the conversion rate is approximately 85%. The degree of sulfamic acid is the lowest, only approximately 75%. It can be found that 180 °C is already a suitable temperature for the reaction, since at this temperature, the reaction esterification rate is higher.

The concentrated sulfuric acid and sodium hydroxide catalytic reaction for 3 h yields an esterification rate difference of approximately 5%. With comprehensive consideration of the experimental safety and esterification rate, sodium hydroxide should be used as a catalyst for oleic acid and ethylene glycol esterification with the optimal reaction temperature being 180 °C.

### 2.3. Esterification of Oleic Acid with Mixed Alcohols

#### 2.3.1. Effect of Temperatures on the Esterification Reaction

**Figure 8:**
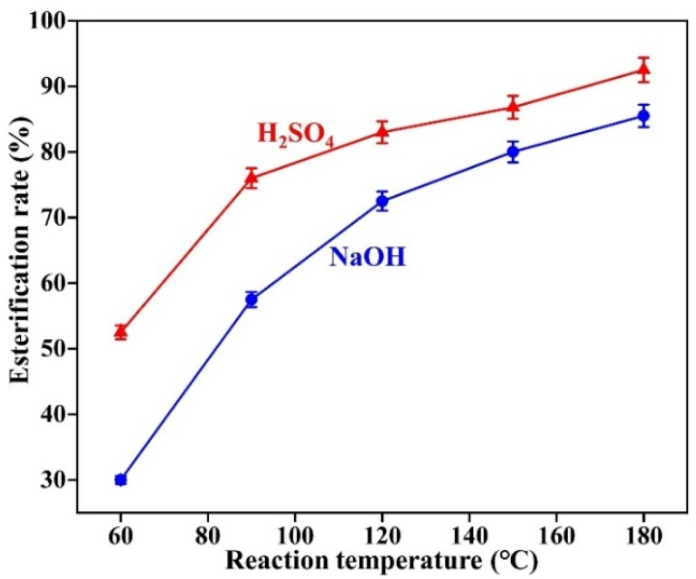
Effect of the reaction temperature on mixed esterification.

The factors affecting the reaction in a mixed esterification experiment are the experimental temperature, the catalyst, the mixing ratio of the two alcohols and the reaction time. According to the previous experiment, the optimal reaction time is 3 h. The mass ratio of methanol to ethylene glycol is set at 1:1. The acid value in the system is measured at the end of the reaction and the esterification rate is calculated according to Equation (4). The results of the experiment are shown in Figure 8.

From Figure 8, it can be concluded that for the mixed esterification of oleic acid with two alcohols, concentrated sulfuric acid is significantly more effective than sodium hydroxide in catalysis. Temperature has a greater influence on the esterification reaction, and as the temperature increases, the esterification rate significantly increases. When the temperature reaches 150 °C, under the catalysis of concentrated sulfuric acid, the esterification rate exceeds 85%. As the temperature continue to increase to 180 °C, the esterification rate reaches 92.5%. Therefore, the mixed esterification temperature should be set at 150 °C.

#### 2.3.2. Effect of the Proportion of Alcohols on Esterification Reactions

**Figure 9:**
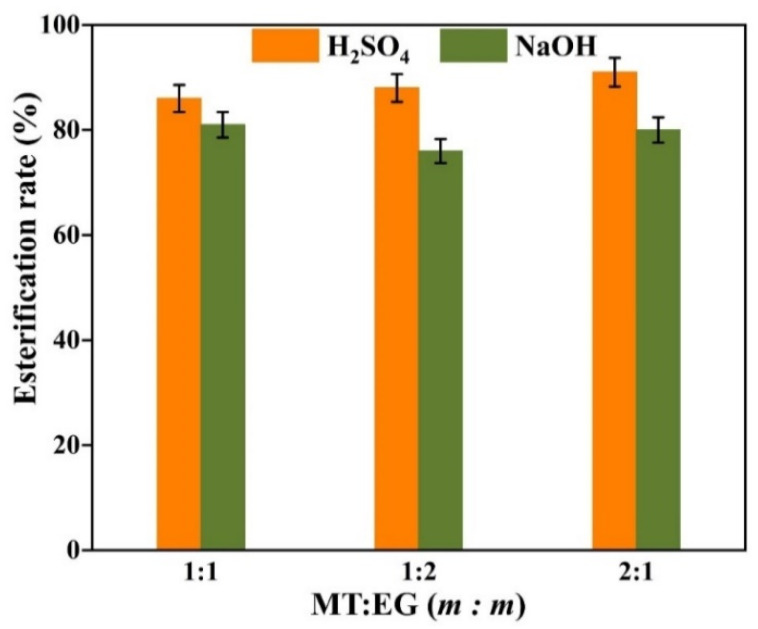
The effect of the proportion of alcohol on the esterification reaction.

With the optimum reaction time set at 3 h and the reaction temperature set at 150 °C, the ratio of the two alcohols was varied to investigate the effect of different ratios of the two alcohols on the experiment. The results of the experiment are shown in Figure 9.

From Figure 9, it can be concluded that the ratio of the two alcohols has less effect on the experimental results. Under the catalysis of concentrated sulfuric acid, the esterification rate is approximately 85%. Catalyzed by sodium hydroxide, the esterification rate is approximately 75%.

On the whole, the best reaction conditions are: reaction time 3 h, reaction temperature 150 °C, and concentrated sulfuric acid as a catalyst. The proportion of alcohol is determined according to the combination of room temperature and high temperature lubricity and foaming rate.

### 2.4. Performance Evaluation of Three Esterification Products

The esterification of oleic acid with methanol, catalyzed by concentrated sulfuric acid, is named as OAE. The esterification of oleic acid with ethylene glycol is named EGO (concentrated sulfuric acid catalyzed product: EGO-1; sodium hydroxide catalyzed product: EGO-2, esterification product (MT:EG = 1:1): MEO-11; esterification product (MT:EG = 2:1): MEO-21; esterification product (MT:EG = 1:2): MEO-12).

#### 2.4.1. FT-IR Analysis of Different Esterification Products

**Figure 10:**
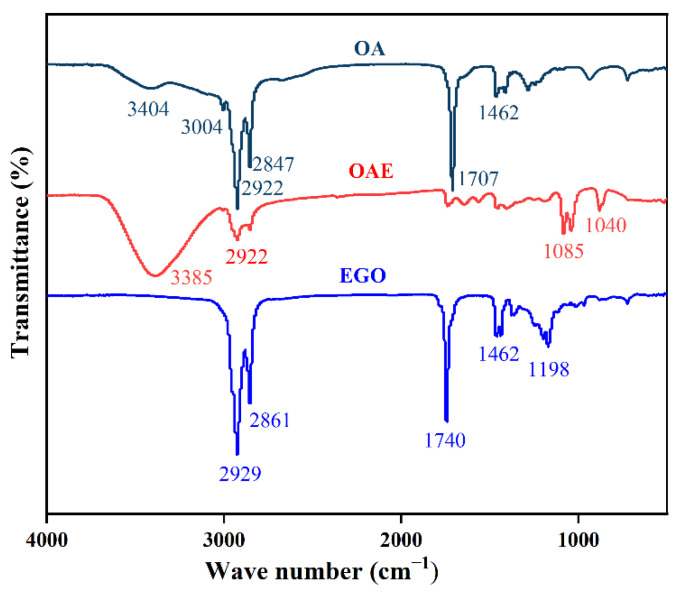
FT-IR of oleic acid and oleate.

Infrared spectroscopy tests were performed on OA and esterification products OAE and EGO, and the test results are shown in Figure 10.

Figure 10 shows the FT-IR of OA esterified by different esterification methods. In FT-IR, –CH_2_ and –COOH correspond to strong absorption peaks at 2922 cm^−1^, 2847 cm^−1^ and 1707 cm^−1^, and –C=C–H corresponds to a peak at 3004 cm^−1^. The plot of the OAE clearly shows the disappearance of the peak (–CH_2_–) of 2922 cm^−1^ and the peak (–COOH) of 1707 cm^−1^, indicating that –CH_2_ is converted into –CH_3_ during esterification, and the occurrence of absorption peaks at 1040 cm^−1^ and 1085 cm^−1^ also indicates the occurrence of this process, with the simultaneous formation of –C–O–C– and –C=O–. In the EGO figure, through the characteristic peaks at 1198 cm^−1^, it can be seen that –COOH is transformed into –C=O–. EGO esterification is insufficient, and there is also a –COOH group corresponding to a strong absorption peak at 1740 cm^−1^.

#### 2.4.2. Basic Performance Analysis of Different Esterification Products

The basic performance evaluation of different esterification products at room temperature is carried out. According to the above experimental methods, the density and fluorescence level of the different oleates are tested, and the test results are shown in Table 1.

According to the test results in Table 1, we compare the indicator requirements. The fluorescence level of all the esters is less than grade 5, which meets the fluorescence level requirement of the base oil of the drilling fluid lubricant. In terms of density variation, the density variation values of various oil esters are all less than 0.08 g/cm^3^ after being added to the base mud, meeting the requirements of the technical index of the liquid lubricant.

#### 2.4.3. Lubricity Analysis of Different Esterification Products

According to the method of 5.6 in the Q/SY 17088-2019 standard, the lubricity of the oil sample used is evaluated, and the evaluation test results are shown in Table 2.

From Table 2, it can be concluded that after the oleate is added to the base mud, the apparent viscosity changes value and the density change value of the base mud is small, which meets the requirements of Q/SY 17088-2016. In terms of lubrication, the performance of OAE at room temperature is excellent, but the lubrication performance is much lower after high temperature hot rolling. EGO-1 and EGO-2 have low lubrication properties at room temperature, however, the performance after high temperature hot rolling is better than the performance at room temperature. MEO-11, MEO-21 and MEO-12 have excellent performance at room temperature, with *Δf* all exceeding 80%. As the hot roll temperature increases, the lubrication performance of MEO-21 becomes better, and *Δf* can reach 89.56% at 180 °C hot rolling. Therefore, MEO-21 can be used as the base oil in the subsequent lubricant formulation process; before selecting a base oil, some key properties need to be checked, and some important properties are kinematic viscosity, temperature stability, and hydrolytic stability [26].

### 2.5. Preparation of High-Performance Lubricants

#### 2.5.1. Compounding of Lubricants

**Figure 11:**
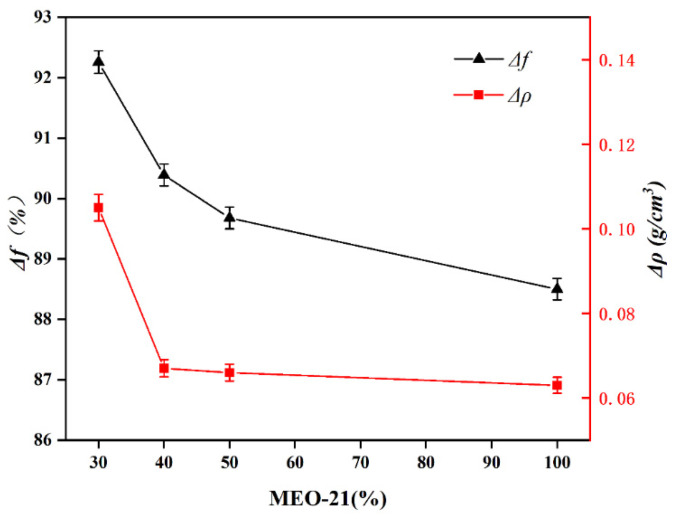
MEO-21 different proportions of test results.

MEO-21 has excellent normal and high-temperature performance, so MEO-21 is used as the base oil, and industrial by-product white oil (WO) and oleic acid (OA) are added to it, and through different ratios, a drilling fluid lubricant with good lubricity and low density variation is obtained, named L-541. The results of the room temperature performance tests corresponding to different proportions of MEO-21 in the system are shown in Figure 11.

From Figure 11, it can be observed that when the proportion of MEO-21 is 40%, the proportion of WO is 50% and the proportion of OA is 10%, the base mud has the smallest *Δρ*, *Δρ* is 0.064 g/cm^3^, as well as has excellent lubricating properties, *Δf* is 90.39%, which meets the requirements of the Q/SY 17088-2016 index. The resulting drilling fluid lubricant was named L-541.

#### 2.5.2. Evaluation of High-Temperature Resistance

**Figure 12:**
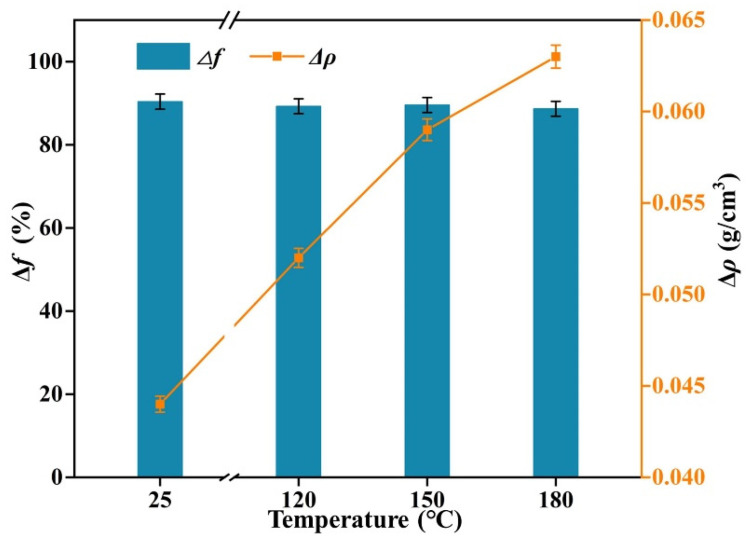
L-541′s high-temperature resistance test results.

The high-temperature resistance of L-541 was evaluated by selecting 120 °C × 16 h, 150 °C × 16 h and 180 °C × 16 h for hot rolling, and evaluating the *Δf* as well as *Δρ* performance indicators before and after the contrasting hot rolling, the experimental results are shown in Figure 12 below.

From Figure 12, it can be concluded that L-541 does not differ much from *Δf* at room temperature and after 16 h of hot rolling at 120 °C, 150 °C and 180 °C, the density is approximately 89%. This shows that L-541 has good high-temperature resistance, at the same time, the density change value is less than 0.08 g/cm^3^, and the density change value meets the index requirements.

#### 2.5.3. Compared with the Same Type of Lubricant

In order to further verify the performance advantages of L-541, the industrial lubricant in use on the market was selected and compared with its performance analysis. The selected comparative samples are the liquid lubricants ZJY-1 and ZJY-2 of CNPC Engineering and Technology Research Institute Co., Ltd. (Beijing, China), liquid lubricants PGCS-1 and HLB of Xinjiang Tuha Oilfield. The comparison test results are shown in Table 3.

From Table 3, it can be concluded that the lubricity of ZJY-1 and ZJY-2 decreased significantly after high-temperature hot rolling, and the lubrication loss was approximately 5%. However, the lubricating performance of L-541 after high temperature hot rolling was stable, higher than ZJY-1, ZJY-2. Compared with Xinjiang Tuha samples HLB and PGCS-1, the performance of L-541 at room temperature and high temperature is better than that of HLB and PGCS-1, the preparation process is more convenient and the price is lower.

## 3. Conclusions

(1) When oleic acid is esterified with methanol, and concentrated sulfuric acid is used as a catalyst while the reaction temperature is 60 °C and the reaction time set at 3 h, the esterification rate is close to 90%. When oleic acid is esterified with ethylene glycol, both concentrated sulfuric acid and sodium hydroxide can be used as catalysts. The reaction temperature is 180 °C, and the reaction is carried out under the catalysis of concentrated sulfuric acid for 3 h, and the esterification rate reaches 90%. Under the catalysis of sodium hydroxide, the esterification rate is approximately 85%.
(2) Under the optimal esterification process, the *Δf* of methyl oleate (OAE) obtained by the esterification of oleic acid and methanol is 85.16% at room temperature, but after hot rolling at 150 °C for 16 h, the *Δf* becomes 72.25%. Lubricity drops significantly after hot rolling at high temperature. When oleic acid is esterified with ethylene glycol, and the prepared ethylene glycol oleate (EGO) has a *Δf* of 80.34% at room temperature, but after 16 h of hot rolling at 150 °C, the *Δf* becomes 82.25% and the lubricating properties rise after hot rolling at high temperature.
(3) When methanol and ethylene glycol are mixed with oleic acid, the optimal reaction conditions are as follows: the reaction time is 3 h, the reaction temperature is 150 °C, and concentrated sulfuric acid is used as the catalyst. The obtained esterification product is named MEO-21. The *Δf* of MEO-21 at room temperature is 86.57%, the *Δf* is 85.25 after hot rolling at 150 °C for 16 h, and the *Δf* is 89.56% after 180 °C hot rolling for 16 h. This mixed esterification method not only simplifies the preparation process, but also the obtained mixed oleic acid ester has excellent lubricity regardless of room temperature or high temperature.
(4) Taking MEO-21 as base oil and compounding with other oils, a high-performance lubricant is prepared, named L-541. After adding L-541 to the base slurry, the base slurry *Δρ* is 0.064 g/cm^3^, and the *Δf* at room temperature is 90.39%, and after 16 h of hot rolling at 120 °C, 150 °C and 180 °C, basically approximately 89%. Compared with the same type of lubricant, L-541 has good high-temperature resistance, stable lubricating performance at room temperature and high temperature, and is a good ester-based lubricant.

## 4. Materials and Methods

### 4.1. Materials

Interventionary white mineral oil (WO) and oleic acid (OA), all of industrial grade were purchased from Jingkai Chemical Factory of Renqiu, Ltd. (Cangzhou, China). Concentrated sulfuric acid, sodium hydroxide, and sulfamic acid are all analytical purities and purchased from Guangdong Thorn Water Technology Co., Ltd. (Guangdong, China). Other reagents include methanol (MT), ethylene glycol (EG), and sodium carbonate anhydrous, which are of analytical purity and were purchased from Beijing Chemical Factory.

### 4.2. Instruments

Extreme Pressure (E-P) Lubrication Device, OFI, (Connecticut, USA). LH-YG1501A Geological Fluorometer, Tianjin Lu hai Petroleum Equipment System Engineering Co. (Tianjin, China). ZNN-D6S Six Speed Rotary Viscometer, Qingdao Hai tong da Special Instruments Company (Qingdao, China). Infrared Spectrometer, Spectrum 100, Perkin Elmer, (Massachusetts, USA), XGRL-4 High Temperature Roller Heating Furnace, Qingdao Chuang Meng Instrument Company (Qingdao, China), Inverter high-speed mixer, Qingdao Chuang Meng Instrument Co., Ltd, (Qingdao, China).

### 4.3. Methods

Preparation of Freshwater Slurry: The method of preparing freshwater-based slurry is referred to in GB/T 20973, 2020., and the detailed preparation method is described in Section 2.3.1 of Ref. [16].

### 4.4. Evaluation Methods

#### 4.4.1. Acid Value Test

The acid value is a measure of the number of free carboxylic acid groups in a compound (fatty acids) or mixture, and it indicates the number of milligrams of KOH required to neutralize 1 g of free fatty acids and can be used as an indicator of the degree of deterioration of fat. The amount of fatty acid can be calculated from the consumption of KOH standard solution as a result of the neutralization reaction between the fatty acid and KOH. The unit is: mg KOH/g.

The acid value of the product is determined according to the method GB 5009.229-2016: Step 1, take 50 mL of ethanol solution in an Erlenmeyer flask, add 2 drops of phenolphthalein indicator, titrate to a slight reddish color with potassium hydroxide standard solution, and note the scale V_1_ of KOH; Step 2, weigh a sample of a certain quality, mark it as m, add it to the aforementioned Erlenmeyer flask, shake well, and let the color fade; Step 3, continue titrating the solution in the Erlenmeyer flask with potassium hydroxide standard solution until it is the same color as the standard color, keeping 30 s without fading as the end point, and record the scale V_2_ of potassium hydroxide in the burette. The formula for calculating the acid value is shown below:

X = {(V_1_ −V_2_) × C_KOH_ × 56.1}/m (2)

In the formula:

X—acid value of the sample being measured (mg KOH/g);
V_1_—scale of KOH after titration in a blank experiment (mL);
V_2_—scale of KOH after sample titration (mL);
C_KOH_—the concentration of KOH standard solution (mol/L);
m—sample quantity (g).

#### 4.4.2. Rheological Performance

The assessment method for apparent viscosity (AV) follows the API standard (API 13B-1, 2009).

AV = 0.5Φ_600_ (3)

In the formula:

AV—apparent viscosity(mPa·s);
Φ_600_—viscometer dial reading at 600 r/min (dia).

#### 4.4.3. Lubricity Performance Test

The evaluation method for the reduction rate of extreme pressure lubrication coefficient (*Δf*) is based on Q/SY 17088-2016. See Section 2.4.1 of Ref. [16] for specific operation steps. Then, calculate its *Δf*, which is calculated as:

*Δf* = (*T*_1_ − *T*_2_)/*T*_1_ × 100 (4)

In the formula:

*Δf*—reduction rate of extreme pressure lubrication coefficient, %;
*T_1_*—extreme pressure lubrication coefficient of the base paste;
*T_2_*—the extreme pressure lubrication coefficient after the base slurry is added to the specimen.

#### 4.4.4. Fluorescence Level Determination of Oil Samples

The fluorescence level of each oil sample was determined according to the 4.3 Fluorescence level determination method in Q/SY 17088-2016: Step 1, take a beaker, wash and rinse with anhydrous ethanol, blow dry and set aside, before adding 20 mL of trichloromethane to the beaker, and then add 1 g (to the nearest 0.01 g) of the oil sample to be tested, shake well and leave to clarify; Step 2, take a part of the clarification solution in a clean test tube, place it under a UV light tester (Beijing Kaihe Water Mirror Instrument Technology Company, Beijing, China)to observe its fluorescence condition, and compare it with the relevant standard series (according to the standard series prepared according to the oilfield crude oil used) to determine its fluorescence level.

**Figure 13:**
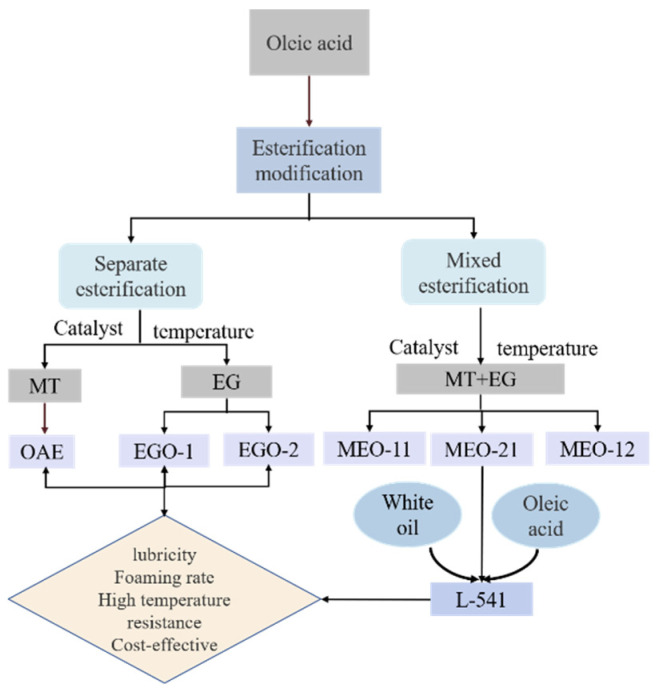
Workflow diagram.

The workflow of this process is shown in Figure 13.

## Figures and Tables

**Table 1:** Performance test results of different oil samples.

Oil Samples	Fluorescence Level, Grade	*ρ*, g/cm^3^	*Δρ*, g/cm^3^
OAE	3	0.99	0.034
EGO-1	3	0.96	0.064
EGO-2	3	0.97	0.054
MEO-11	3	0.99	0.033
MEO-21	3	0.96	0.063
MEO-12	3	0.99	0.033

**Table 2:** Lubricity test results of different oleate esters.

Evaluation Methods	Q/SY	OAE	EGO-1	EGO-2	MEO-11	MEO-21	MEO-12
AV, mPa·s	5≤	4	4.5	5	5	4.5	5
*Δρ*, g/cm^3^	0.08≤	0.034	0.064	0.054	0.033	0.063	0.043
*Δf* (Room temperature), %	≥85	85.16	80.34	81.16	84.46	86.57	82.26
*Δf* (120 °C × 16 h Hot Rolling), %		82.34	80.15	85.35	83.34	85.15	83.35
*Δf* (150 °C × 16 h Hot Rolling), %		72.25	82.25	82.46	85.25	85.25	82.46
*Δf* (180 °C × 16 h Hot Rolling), %		70.59	77.56	82.57	79.59	89.56	80.57

**Table 3:** Comparative test results of different lubricants.

Evaluation Method	Comparison Samples				
	L-541	ZJY-1	ZJY-2	PGCS-1	HLB
Fluorescence level, grade	3	3	3	4	4
AV, mPa·s	3	4	3.5	4	5
*Δρ*, g/cm^3^	0.064	0.064	0.054	0.064	0.084
*Δf* (Room temperature), %	90.39	91.27	92.78	86.16	85.09
*Δf* (120 °C × 16 h), %	89.27	89.65	91.34	87.21	87.06
*Δf* (150 °C × 16 h), %	89.57	88.34	87.45	87.26	86.13
*Δf* (180 °C × 16 h), %	88.58	84.21	87.35	86.45	86.60
Price, CNY/ton	7000	8000	8500	8000	7000

## Data Availability

The data are contained within the article.
